# Being on the Same Page: Ongoing Adjustment of the Therapeutic Contract When Treating Pathological Narcissism and Narcissistic Personality Disorder

**DOI:** 10.62641/aep.v54i4.2305

**Published:** 2026-08-15

**Authors:** Giancarlo Dimaggio, Gabriele De Gabrielis, Angus MacBeth, Felix Inchausti

**Affiliations:** ^1^Center for Metacognitive Interpersonal Therapy, 00193 Rome, Italy; ^2^Clinical and Applied Psychology, University of Edinburgh, EH8 9AG Edinburgh, Scotland, UK; ^3^Department of Mental Health, Psychotherapy Research and Practice Unit, San Pedro University Hospital, 26006 Logroño, Spain

“The devil is in the details” in treating pathological narcissism (PN) and narcissistic personality disorder (NPD). Countering historical pessimism, effective interventions exist [1, 2, 3] but with no randomised clinical trials or guidelines. However, there are principles which offer a clinician-ready roadmap towards effective treatment [2, 4, 5]: (a) helping patients identify goals and therapy direction, promoting agency; (b) techniques to resist engaging in power struggles with patients; (c) considering treatment disruptive behaviours, e.g., missed sessions; (d) fostering self-reflective capacity; (e) reducing maladaptive ideas about self and others; and accessing more positive ideas, e.g., promoting self-acceptance, enabling patients to pursue meaningful life goals driven by curiosity and connection, irrespective of social rank motives [4, 6, 7]; (f) promoting agency and behavioural change to counteract narcissistic tendencies towards passivity or reactivity to external events; (g) helping process losses and setbacks and interrupting tendencies to divert the mind from painful, negative feelings through intellectualization, minimisation, or risky behaviours (e.g., substances, seduction) [5, 8].

The struggle to articulate concrete, shared therapeutic goals in NPD/PN precipitates patients’ dislike of interruption, generating frustration and constriction when challenged. Negotiated agreement on concrete, pragmatic and measurable goals is required to licence therapeutic tasks. These inform the activities patients need to realise positive goals. Without this, therapeutic progress atrophies.

Accordingly, drafting a contract when commencing therapy and continuously updating as therapy progresses is fundamental [4, 5, 9]. A contract denotes the steps for therapist-patient agreement on goals, making explicit that without committing to tasks therapeutic goals will remain elusive, and that patients consciously choose to commit to these tasks. How then to agree tasks and goals in treating PN/NPD? We propose that precision is required for effective intervention, ensuring patient and therapist are “on the same page”. Here we outline a step-by-step procedure to enable clinicians and patients to identify the absence of a contract and then broker agreement.

The following factors compromise PN/NPD’s capacity to set and realise therapeutic goals:

1. External world focus: Blaming others for their problems, such as complaining that colleagues don’t recognise their incredible qualities, or the world does not recognise the wrongs they suffered. This prevents them pursuing well-being goals.

2. Comorbidity and problematic behaviours: These reflect maladaptive valued goals—“Substances make me feel creative”; “Why should I eat normally? Do you want me to be a fat loser?”

3. Repetitive thinking as coping: “If I stop worrying, I risk failure, rejection and feeling guilty”; “If I give up competing, I will fail” [4].

4. Absence of therapeutic relationship: Maladaptive interpersonal patterns lead patients to perceive therapists as potentially critical, incompetent, intrusive, coercive, manipulative or omnipotent. This may lead the individual to close off, becoming distrustful and self-censor their narrative [1, 10, 11].

5. Helplessness, diminished agency and hopelessness; “Why talk about my inner world if nothing can be done?”; “I understand better what makes me feel bad, but then what?”. When people with PN step outside the social rank motive, they shut down, lacking an inner motivation. This precipitates lack of agency, inability to feel their own desires, and comprehending that these can be pursued [4].

We propose a step-by-step procedure to develop tasks and objectives with patients presenting with PN/NPD, addressing the above problems (Fig. 1).

**Fig. 1.**
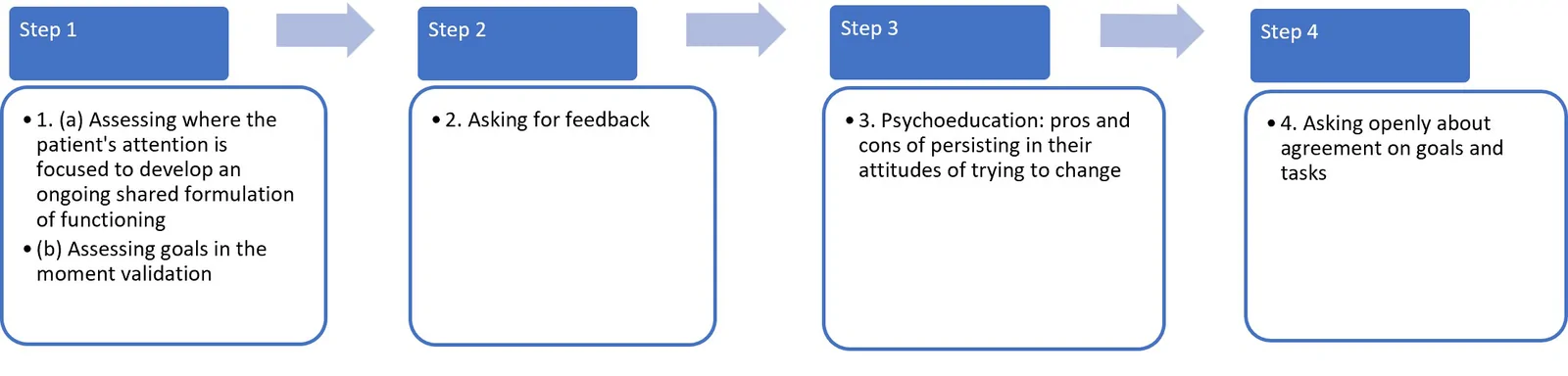
**Steps to ensure the continuous adjustment of the therapeutic contract in the treatment of Pathological Narcissism and Narcissistic Personality Disorder**. Step by step procedures. This figure illustrates the four steps to ensure the continuous adjustment of the therapeutic contract in the treatment of PN and NPD. PN, pathological narcissism; NPD, narcissistic personality disorder.

## Step 1

### Assessing Attentional Focus to Develop an Ongoing Shared Formulation of Functioning

Therapy requires continuous monitoring of the patient’s attentional focus, to inform an ongoing shared formulation. Monitoring means attending to the patient’s mental attitude and asking specific questions. Inwardly, clinicians ask themselves whether, in the episode the patient is recounting, is attention focused on the internal or external world. Is the patient just blaming others? The therapist can be explicit: “You are frustrated about your work situation, although I realize that you are focused on describing how others are stupid or unsupportive. I fully understand, but I am struggling to understand how we translate this into therapy goals that we can share and work with”.

Here the clinician assesses Factors 4 (therapeutic relationship absence) and 5 (hope) from the framework. Does the patient have positive ideas about the therapist - someone they can trust, ask for support, who is present and competent? Or do they consciously or implicitly perceive the therapist as incompetent, neglecting or un-understanding?

### Assessing Patient Goals in the Moment

Are patients striving for health or enacting coping strategies? Are these maladaptive behaviours both counterproductive and egosyntonic? This information can update the shared formulation.

Accordingly, clinicians must identify the motivational systems and goals underlying patient narratives [4]. When patients complain about a behavioural outcome, was the behaviour intended to protect from guilt [7], shame or sadness, or was it a conscious, deliberate choice they are unwilling to give up? For instance, are patients exhausted by the overexercise, perfectionism, repetitive thinking, but still staying with their lover, engaging in disordered eating, or sex addiction? The calculation is whether the emotional benefit of addressing these intentions outweighs the distress. Intervention at this level is purely psychoeducational, not motivational. Rather than immediately promote changing, for which patients may be unready, it enables them to become aware of the intentions underlying their mental attitudes, e.g., perfectionism or rumination as cognitive stances, with corresponding links to behaviours.

## Step 2

### Seeking Feedback

After formulating, clinicians assess whether patients agree with the description. Is the formulation sufficiently accurate and experientially resonant in describing the patients’ experience? Simple questions are key: “Does that sound right? Can you see yourself in the way I’ve described you?”

Therapists often overlook this step, assuming that after describing patients’ functioning, the latter has accepted it, with therapy proceeding to changing patterns. However, patients often disagree because they remain driven by their schemas and biases. Disagreements signals include silence, subject changes, or pleasing the therapist, in the absence of consistent nonverbal change markers. Clinicians should further explore the patients’ reactions, giving space to discuss disagreement.

Individuals with narcissism often openly express criticism, which requires understanding both the momentary processes in the therapy relationship and the presence of formulation errors. Clinicians need to be guided by curiosity and openness to revising formulations, either until ruptures have been resolved and/or the formulation accurately mirrors the patients’ experience.

## Step 3

### Psychoeducation: Weighing up Costs and Benefits of Change

PN/NPD patients tend to persist in their original coping behaviours (e.g., perfectionism, verbal aggression, substance abuse) to either maintain self-esteem or to experience novelty and creativity [4, 12]. This maintains frustration and suffering, but once they have understood their functioning, the question moves to: are they willing to change? Clinicians must not assume this is the case.

Here, clinicians need to resist the urge to force change or to pressure towards a perceived “healthy” option. Building motivation is key, alongside psychoeducation. This involves offering an accurate, detailed portrait of possible future scenarios, explicitly outlining alternative the short- and long-term consequences of behavioural trajectories. Clinicians need to be calm and nonjudgmental, balancing costs and benefits of change against those of continuing with current coping. One can acknowledge substance use or risky behaviours feel “cool”, with short-term dopaminergic pleasure counteracting emptiness and numbness. Competition is normal, calling out other people’s perceived mistakes is rewarding. Conversely, reducing problematic behaviours is sought by the patient, but the cost is an increase (albeit hopefully transient) in anxiety, shame, guilt or sadness. Remaining validating and empathetic, clinicians present possible paths, outlining their relative positive and negative implications. A therapist can, and is obliged to, express a preference for the path towards health but cannot impose this view. Otherwise, patients may feel forced, triggering potential opposition and rebellion, leading to stagnation or drop-out.

## Step 4

### Openly Negotiating Agreement on Goals and Tasks

After psychoeducation, it is important to avoid jumping straight into action. Clinicians must first assess whether, after reflecting on costs and benefits of each path, PN/NPD patients have contracted to explore behaviour and attitudinal change. Have they fully evaluated all options and genuinely deciding to try and change something by the next session?

Clinicians should adopt a wait-and-see attitude, avoiding a “push” towards health. In the moment clinicians can explore what the patient is deciding and ensure they have considered all implications. For instance, do they accept giving more attention to their children reduces time for work or dating?

If patients talk about persisting with old habits, this can prompt renegotiating the contract or revisiting therapy goals. Therapists never force patients to change and can accept deciding to abandon a goal, but some reasonable goal is always required for patients want to benefit from treatment. This openness prevents patient disappointment, frustration and criticism.

Treating PN/NPD remains challenging and there is the need for both empirical testing of manualized approaches and of structured, rationale and replicable procedures [10]. Supporting this, we have outlined a step-by-step procedure for dealing with contract breaches, recognising absence of a contract and modelling shared-decision making until reasonable, realistic, concrete goals can be agreed upon and patients commit to therapy-relevant tasks.

These procedures could be empirically tested through outcome and process research, evaluating their contribution to therapeutic change and repairing alliance ruptures. This could reduce attrition, offering patients with PN/NPD more effective treatment options.

## Availability of Data and Materials

Not applicable.
